# Specialized phosphate transport is essential for *Staphylococcus aureus* nitric oxide resistance

**DOI:** 10.1128/mbio.02451-23

**Published:** 2023-11-08

**Authors:** Amelia C. Stephens, Srijon K. Banerjee, Anthony R. Richardson

**Affiliations:** 1 Department of Microbiology and Molecular Genetics, University of Pittsburgh, Pittsburgh, Pennsylvania, USA; New York University School of Medicine, New York, New York, USA

**Keywords:** phosphate, *S. aureus*, nitric oxide, pathogenesis

## Abstract

*Staphylococcus aureus* is a major human pathogen capable of causing a variety of diseases ranging from skin and soft tissue infections to systemic presentations such as sepsis, endocarditis, and osteomyelitis. For *S. aureus* to persist as a pathogen in these environments, it must be able to resist the host immune response, including the production of reactive oxygen and nitrogen species (e.g., nitric oxide, NO·). Extensive work from our lab has shown that *S. aureus* is highly resistant to NO·, especially in the presence of glucose. RNA-seq performed on *S. aureus* exposed to NO· in the presence and absence of glucose showed a new system important for NO· resistance—phosphate transport. The phosphate transport systems *pstSCAB* and *nptA* are both upregulated upon NO·-exposure, particularly in the presence of glucose. Both are key for phosphate transport at an alkaline pH, which the cytosol of *S. aureus* becomes under NO· stress. Accordingly, the Δ*pstS*Δ*nptA* mutant is attenuated under NO stress *in vitro* as well as in macrophage and murine infection models. This work defines a new role in infection for two phosphate transporters in *S. aureus* and provides insight into the complex system that is NO· resistance in *S. aureus*.

**IMPORTANCE:**

*Staphylococcus aureus* is a bacterial pathogen capable of causing a wide variety of disease in humans. *S. aureus* is unique in its ability to resist the host immune response, including the antibacterial compound known as nitric oxide (NO·). We used an RNA-sequencing approach to better understand the impact of NO· on *S. aureus* in different environments. We discovered that inorganic phosphate transport is induced by the presence of NO·. Phosphate is important for the generation of energy from glucose, a carbon source favored by *S. aureus*. We show that the absence of these phosphate transporters causes lowered energy levels in *S. aureus*. We find that these phosphate transporters are essential for *S. aureus* to grow in the presence of NO· and to cause infection. Our work here contributes significantly to our understanding of *S. aureus* NO· resistance and provides a new context in which *S. aureus* phosphate transporters are essential.

## INTRODUCTION

*Staphylococcus aureus* is a human skin pathogen capable of causing both mild and severe infections. Mild and manageable presentations include skin and soft tissue infections, whereas severe cases can result from the spread of the infection throughout the body to cause endocarditis, pneumonia, osteomyelitis, and/or sepsis (1–6). The diversity of infections caused by *S. aureus* is a unique facet of this bacterium’s pathogenesis and can be attributed to several different causes including the wide array of toxins and proteases expressed during infection (7). These factors afford *S. aureus* the ability to resist antibody-mediated phagocytosis, to kill and escape from successful phagocytes, and the ability to resist host immune radicals such as reactive oxygen and nitrogen species (8).

Our lab has extensively studied the response of *S. aureus* to host nitric oxide (NO·) (9–16). The primary targets of host NO· are transition metals such as the iron molecules in heme centers and iron sulfur clusters and cytosolic thiols such as cysteine (17, 18). These motifs are over abundant in specific metabolic pathways including the TCA cycle and the electron transporter chain (ETC). Thus, *S. aureus* adopts a metabolic scheme that relies less on these pathways and more on glycolytic substrate-level phosphorylation. To achieve this, *S. aureus* requires robust import of glucose, high levels of glycolytic flux, and a highly active lactate dehydrogenase (Ldh1) (9, 11). Given the importance of high flux through this pathway for growth in the presence of NO·, many of the key reactions are carried out by seemingly redundant enzymes/transporters. For instance, *S. aureus* recently acquired two additional glucose transporters (*glcA* and *glcC*) to facilitate rapid uptake of glucose during infection (13). The pathogen also acquired an additional lactate dehydrogenase (*ldh1*) to account for the redox imbalance that occurs due to NO·-mediated inhibition of the ETC (9). Another important facet of the NO·-resistant metabolic state is for the pathogen to maintain adequate inorganic phosphate levels to support the sole energy-producing substrate-level phosphorylation in glycolysis. To this end, there are three systems that are used for inorganic phosphate transport in *S. aureus* (19). Recently, a thorough study demonstrated that there are different conditions in which each of these transporters are vital for *S. aureus* growth (19). The first, and most complex, system is the PstSCAB system, an ABC transporter that uses PstS as a shuttle for inorganic phosphate (20–23). This system has the highest affinity for inorganic phosphate, and its expression is induced when phosphate is limited. Second, the PitA system is dependent on proton-motive force and is the primary form of phosphate transport (20, 23, 24). This system is particularly important when extracellular inorganic phosphate is plentiful. Finally, the NptA system is a sodium-dependent phosphate antiport system, which was recently “reacquired” by *S. aureus* from some staphylococcal relative (Fig. S1) (19). PitA and NptA are both known to be influenced by pH. (25)

**Fig 1:**
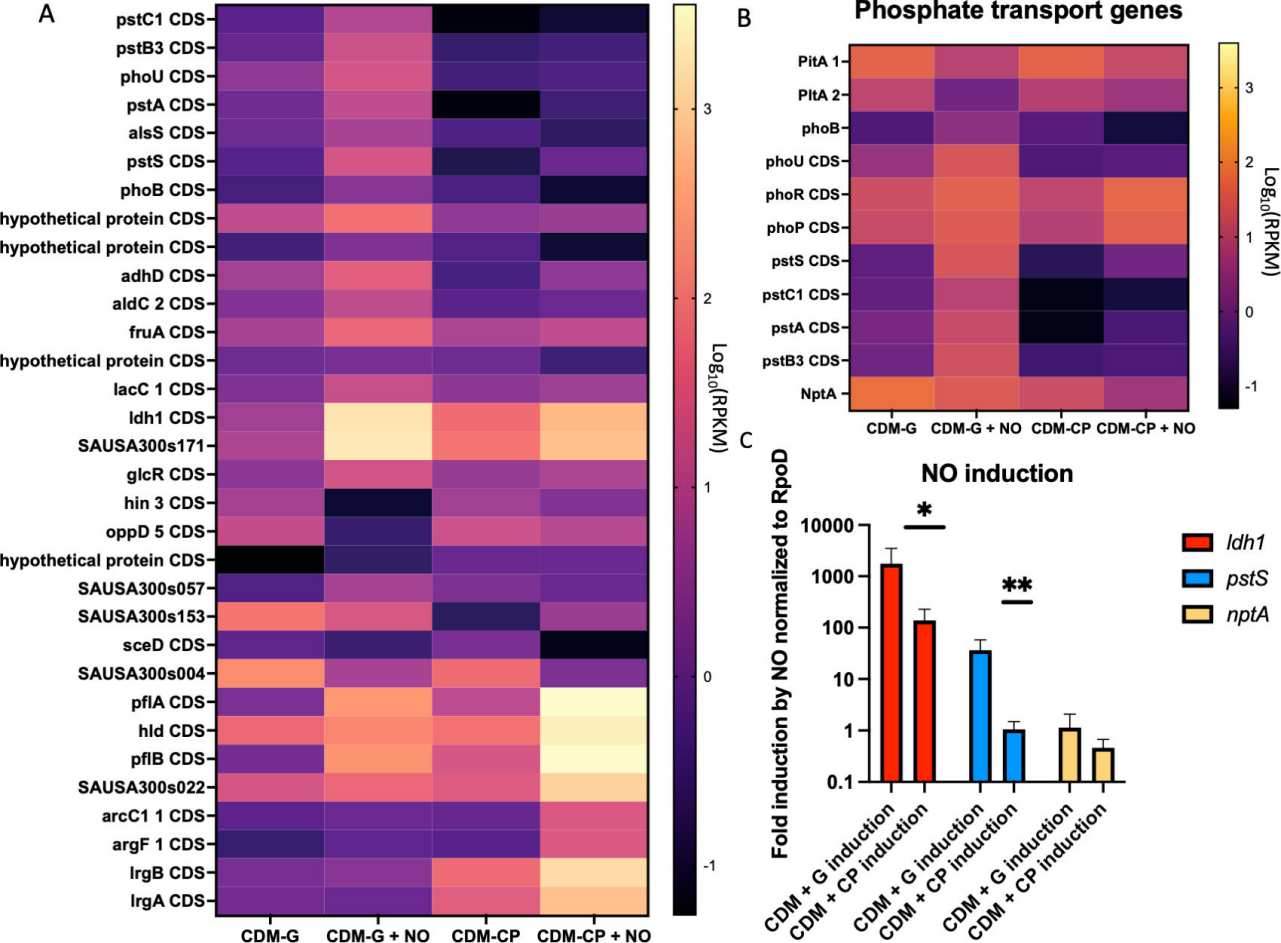
Phosphate transporters *pstSCAB* and *nptA* are upregulated in glucose. Cultures of WT LAC were grown to an OD of 0.5, and RNA samples were taken. 10 mM DETA-NO was added to the remaining culture, and further samples were taken after 15 minutes. The resulting RNA was sequenced, and the results were parsed for differential expression between CDM-G and CDM-CP, as well as genes that are upregulated by NO in one media or the other (**A**). The top results prompted us to look at phosphate transporter expression in this data set (**B**). We independently confirmed the expression of *pstS* and *nptA* with qRT-PCR in triplicate, using *ldh1* as a control for NO induction (**C**). Statistics: unpaired *t*-tests of genes * = *P* < 0.05, ** = *P* < 0.01.

The *ldh1* gene is controlled by a redox-sensing repressor known as Rex (26). Rex binds to NADH when levels get too high, causing the repressor to lose DNA-binding affinity and derepressing the entire regulon. However, redox imbalance is not the only signal that affects *ldh*1 expression. Ldh1 is more highly expressed during NO· stress in the presence of glucose than in its absence (26). This and other factors make glucose essential to *S. aureus* in the presence of host NO·. However, the mechanism of glucose-dependent control of *ldh*1 is entirely unknown. Typical regulators of carbon catabolite repression, like CcpA, are not responsible for the direct regulation of *ldh*1 in the presence of glucose (26). Here, we employed RNA-Seq to define the set of genes that, like *ldh*1, are induced by NO· to a much higher level in the presence of glucose than in its absence. The highest differentially induced genes encoded the Pst and NptA phosphate transport systems (Fig. 1), which have been shown to be required for growth under conditions we hypothesized would be relevant to NO· resistance. We also assess the importance of these transporters in the presence of host NO· both *in vitro* as well as *in vivo* and show their requirement for efficient glycolytic substrate level phosphorylation and ATP production.

## RESULTS

### Phosphate transporters are transcriptionally upregulated by NO, particularly in the presence of glucose

We conducted RNA-sequencing on samples of wild-type (WT) *S. aureus* strain LAC grown to early exponential phase in chemically defined minimal media (CDM) containing two different carbon sources—(i) 0.5% glucose and (ii) a combination of 0.5% casamino acids and 0.5% pyruvate. RNA was isolated from each of these cultures at OD_660_ = 0.5, and the NO donor DETA-NO was added to the remaining cultures at a concentration of 10 mM for an additional 15 minutes. Afterward, RNA was isolated from each of these cultures as well. We analyzed all data sets for significantly regulated genes—that is, genes whose RPKM is more than two SDs removed from the average—and present them in Table S1. Fig. 1A shows the relative expression of the top genes differentially regulated by NO and significantly different between CDM-G + NO and CDM-CP + NO. Interestingly, while *ldh1* does come out of this analysis, it is not the gene most differentially regulated. The top genes from this analysis were all related to the phosphate transport system *pstSCAB* and its regulator *phoU*. We also see the differential regulation of a few hypothetical proteins, some sRNAs, and other genes (Fig. S2).

*S. aureus* has three phosphate transport systems*—pstSCAB*, *nptA*, and *pitA*, all of which were assessed for expression under these conditions (Fig. 1B). Using qRT-PCR, we validated findings from the RNA-Seq data set. We found no difference in *pitA* expression in either medium with/out NO (Fig. S3A). We also found that basal *nptA* and *pstS* expression was higher in the presence of glucose than in its absence (Fig. S3B). Additionally, we assessed the CDM-G and CDM-CP used in the RNA-seq experiment for phosphate content. There is no difference in phosphate content between these two media (Fig. S3C). We independently confirmed that the expression of *pstSCAB* is induced by NO to a much higher level in the presence of glucose than in the absence, while *nptA* is not induced by NO (Fig. 1C). Additionally, as with *ldh*1, this glucose responsiveness is neither dependent on CcpA nor was it dependent on phosphate responsive regulators such as PhoU or PhoPR (Figure S4). Thus, it seems as though *pstSCAB* responds to both NO and glucose, while *nptA* responds only to glucose, both by unknown mechanisms.

### Phosphate transporters PstSCAB and NptA are vital for growth under NO· stress

**Fig 2:**
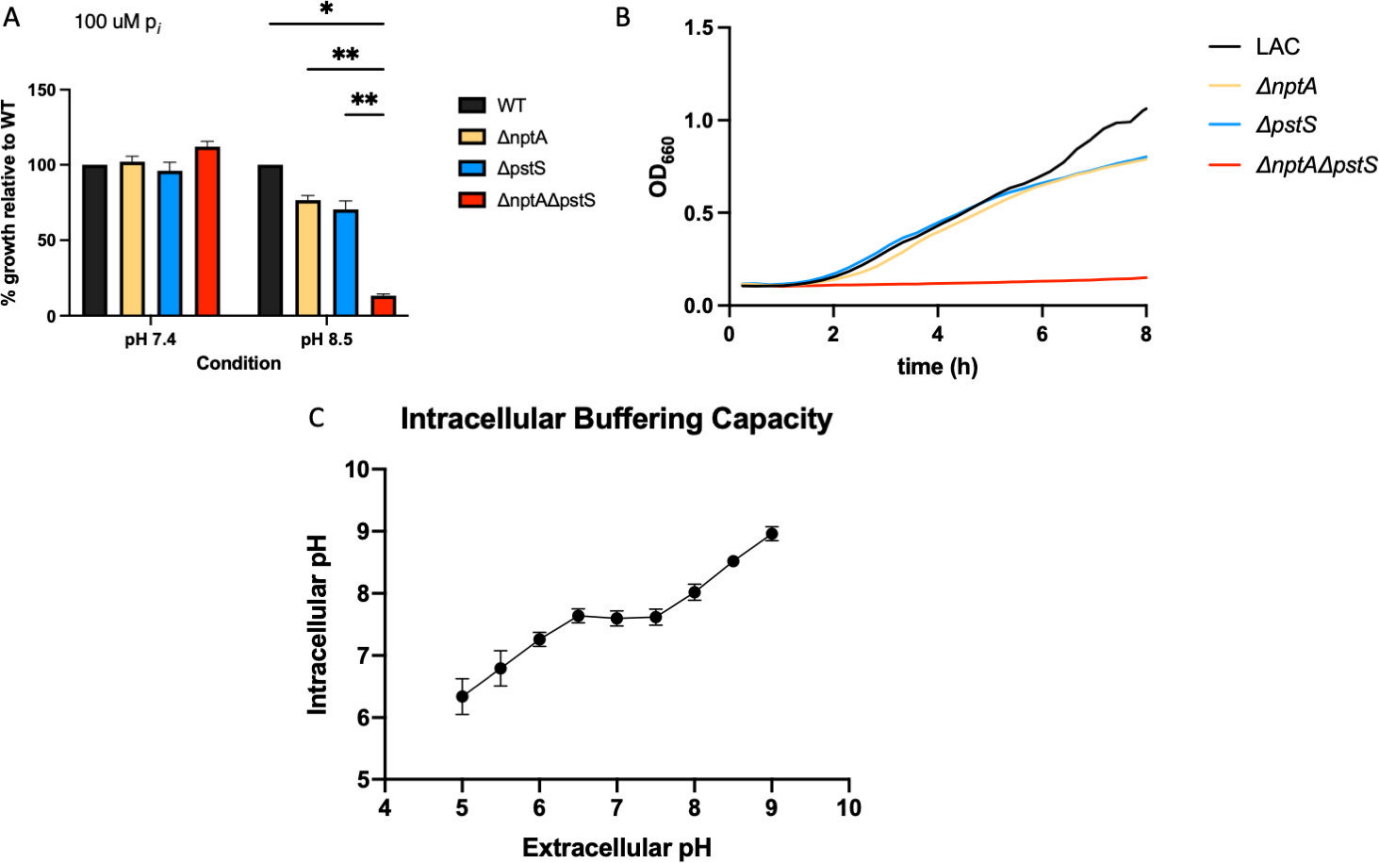
Phosphate transport via NptA or PstSCAB is needed for growth in alkaline conditions. WT LAC, Δ*nptA*, Δ*pstS*, and Δ*nptA*Δ*pstS* were grown in phosphate limiting (100 µM p*_i_*) CDM + G. The OD of these strains at 8 h were normalized to WT LAC and graphed in panel **A**. A representative growth curve is in panel **B**. The intracellular pH of WT LAC growing in various pH CDM + G was determined and graphed as a function of extracellular pH (**C**). Statistics: two-way ANOVA with Tukey’s multiple comparisons. * = *P* < 0.05, ** = *P* < 0.01.

Based on our RNA-Seq data, we developed individual mutants in *pstS* and *nptA*. We also created a double mutant, Δ*nptA*Δ*pstS*. A previous study characterizing the three phosphate transport systems in *S. aureus* demonstrated that there is a need for *nptA* or *pstSCAB* in high pH conditions (19). We grew our mutants in a low phosphate (100 µM p*_i_*) CDM + glucose at a pH of 7.4 and 8.5 and found that the double mutant Δ*nptA*Δ*pstS* exhibited significant lag as compared to both WT LAC and the single mutant counterparts specifically under alkaline conditions (Fig. 2A and B). We did not observe a growth defect in the Δ*nptA*Δ*pstS* mutant when grown in our unaltered CDM + glucose or high phosphate (10 mM p*_i_*) CDM + glucose at pH 7.4 or 8.5, indicating that supplementation of additional free phosphate can alter this phenotype (data not shown). Genetic complementation of both *nptA* and *pstSCAB* was performed in the Δ*nptA*Δ*pstS* mutant, demonstrating that the expression of either phosphate transport system is sufficient to restore growth to the Δ*nptA*Δ*pstS* mutant at a high pH (Fig. S5A and B).

Our lab has previously demonstrated that the intracellular pH of a *S. aureus* strain growing in the presence of NO is ~8.5 (15). We tested whether *S. aureus* defends the cytoplasmic pH of the cell against the external pH of the media (Fig. 2C). We found that while *S. aureus* does defend against extracellular acidity (internal pH ~7 at an external pH of ~5.5), it does not defend against extracellular alkalinity (Fig. 2C). Thus, at an extracellular pH of ~8.5, *S. aureus* exhibits an intracellular pH of ~8.5—virtually identical to the internal pH of *S. aureus* in the presence of NO·.

**Fig 3:**
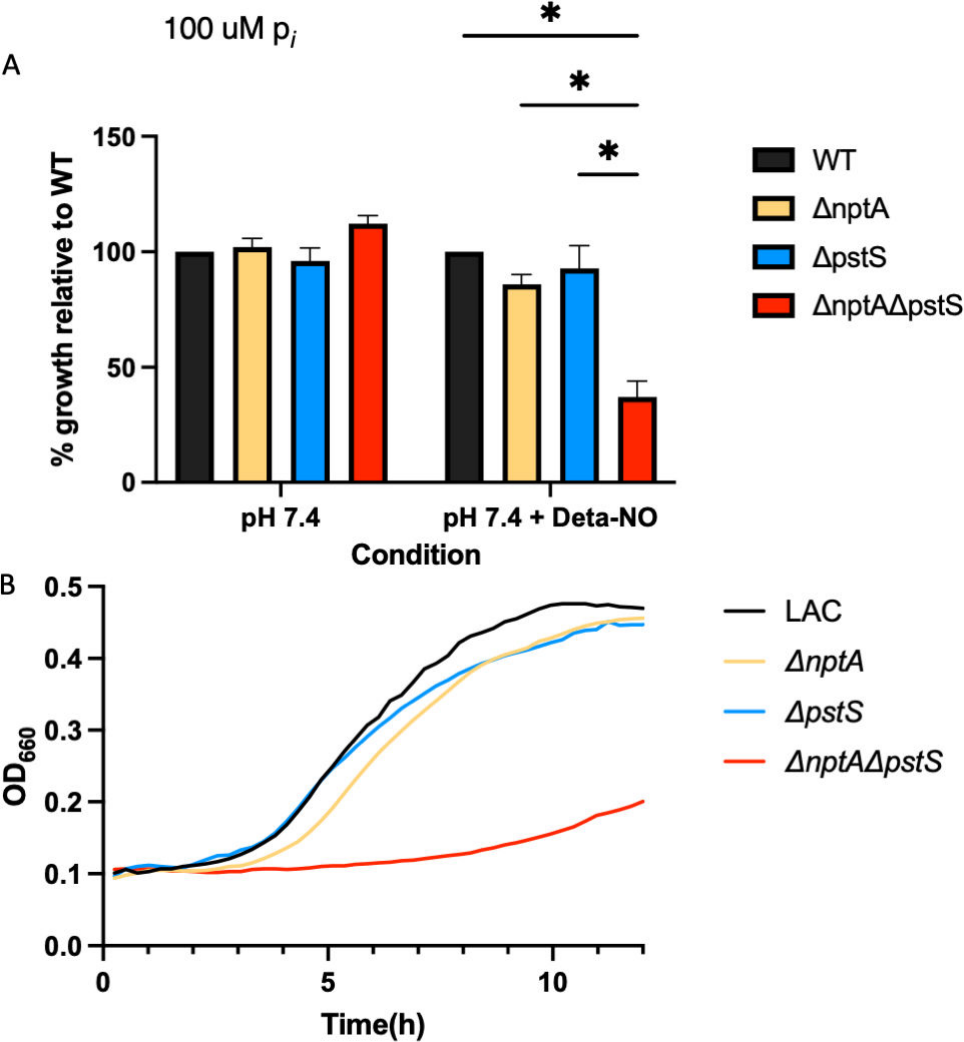
Phosphate transport via NptA or PstSCAB is needed for growth under NO stress. WT LAC, Δ*nptA*, Δ*pstS*, and Δ*nptA*Δ*pstS* were grown in phosphate limiting (100 µM p*_i_*) CDM + G and exposed to 10 mM DETA-NO from inoculation. The OD of each of these strains at 8 h was normalized to WT LAC and graphed in panel **A**. A representative growth curve is in panel **B**. Statistics: two-way ANOVA with Tukey’s multiple comparisons. * = *P* < 0.05.

Therefore, we tested the impact of NO· on our phosphate transport mutants. Grown in the same phosphate limiting (100 µM p*_i_*) CDM-glucose, with 10 mM DETA-NO donor added at inoculation of cultures, the Δ*nptA*Δ*pstS* mutant displays a similar lag in growth as in alkaline conditions (Fig. 3). Again, there is no defect in growth for the single mutants or a growth defect in non-altered CDM-G or high-phosphate (10 mM p*_i_*) CDM-G. Genetic complementation with *nptA* or *pstSCAB* is sufficient to restore unaltered growth of the Δ*nptA*Δ*pstS* mutant in the presence of NO·. Taken together, we conclude that the intracellular pH of *S. aureus* dictates which phosphate transporters are necessary for growth under limiting phosphate. We also find that in the presence of NO·, either *pstSCAB* or *nptA* is necessary for *S. aureus* growth.

### Phosphate transport mutants have lowered ATP

**Fig 4:**
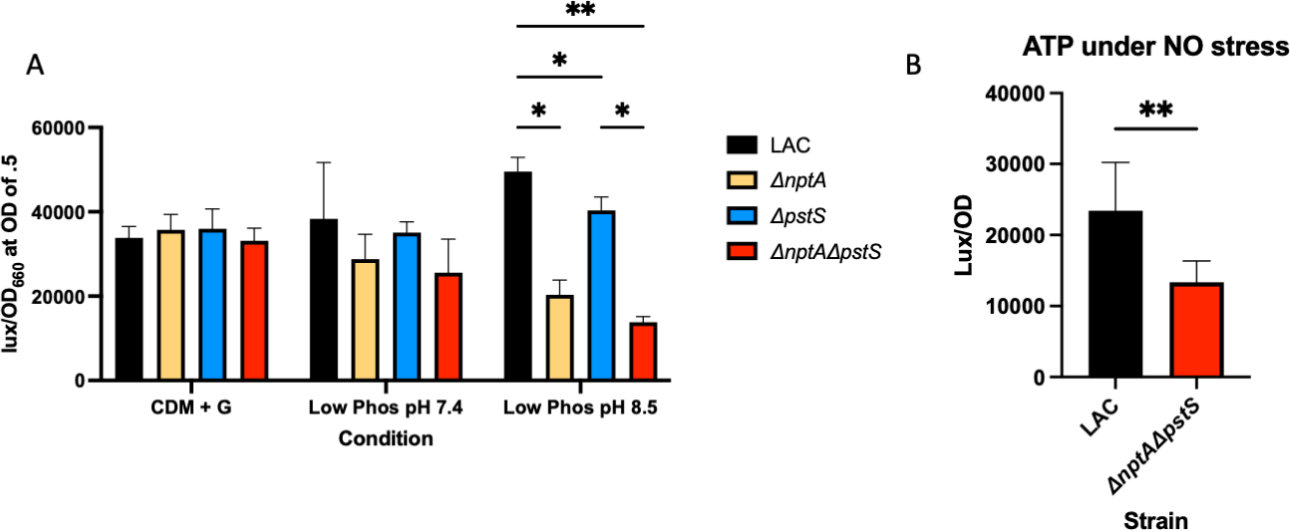
Intracellular ATP is lowered in a Δ*nptA*Δ*pstS* double mutant as compared to WT. WT LAC, Δ*nptA*, Δ*pstS*, and Δ*nptA*Δ*pstS* were grown in various CDM + G, and intracellular ATP was determined for each strain and normalized to the strain’s OD_660_ at that time point. (**A**) The ATP of each strain non-phosphate limiting CDM + G, low phosphate (100 µM p*_i_*) CDM + G at pH 7.4, and low phosphate (100 µM p*_i_*) CDM + G at pH 8.5 (*n* = 3). (**B**) The ATP of WT LAC and Δ*nptA*Δ*pstS* grown in low phosphate CDM + G at pH 7.4 and exposed to 10 mM DETA-NO from inoculation (*n* = 8). Statistics: (**A**) two-way ANOVA with Tukey’s multiple comparisons; (**B**) unpaired *t*-test, * = *P* < 0.05, ** = *P* < 0.01.

While inorganic phosphate plays a role in several cellular functions in the bacterium, a major one is the formation of ATP via glycolytic substrate-level phosphorylation. Glyceraldehyde-3-phosphate dehydrogenase (GAPDH) incorporates inorganic phosphate into glyceraldehyde-3-PO_4_^−^ to yield 1,3-bisphosphoglycerate. This assimilated phosphate will be used to generate ATP in the next step, allowing for the incorporation of exogenous phosphate into the ATP pool. GAPDH requires adequate intracellular inorganic phosphate levels to function efficiently. To this end, we tested our Δ*nptA*, *ΔpstS*, and Δ*nptA*Δ*pstS* mutants for intracellular ATP levels in CDM + glucose, and phosphate limiting (100 µM p*_i_*) CDM + glucose at pH 7.4 and 8.5. We found that while there was no difference in ATP levels between the mutants and WT in regular CDM-glucose or pH 7.4 phosphate limiting CDM-glucose, there was a significant reduction in ATP in the Δ*nptA*Δ*pstS* strain at a high pH (Fig. 4A). Interestingly, there also appears to be a defect in ATP levels in a Δ*nptA* single mutant as well—but no phenotype was displayed in either condition by this mutant (Fig. 2 and 3). This indicates that the ATP defect may not be directly contributing to the growth defect we observe above, and the phosphate import by NptA is more vital for cellular functions besides incorporation into ATP. We also tested the impact of NO· on ATP levels in WT LAC and Δ*nptA*Δ*pstS*. We found, again, significantly reduced ATP levels in this mutant under NO stress (Fig. 4B). This link between phosphate transport and ATP levels indicates that these transporters are vital for glycolysis and the synthesis of ATP, something that is extremely important in NO· stressed cells, as glycolysis becomes the primary form of energy generation under these conditions. This link is possibly the reason for the increased expression of these two phosphate transporters observed in the RNA-sequencing experiment. However, we still do not have a molecular mechanism behind the glucose-dependent expression of *pstSCAB*, *nptA*, or *ldh1*.

### *In vivo* phenotypes indicate PstSCAB and NptA are vital for infection

**Fig 5:**
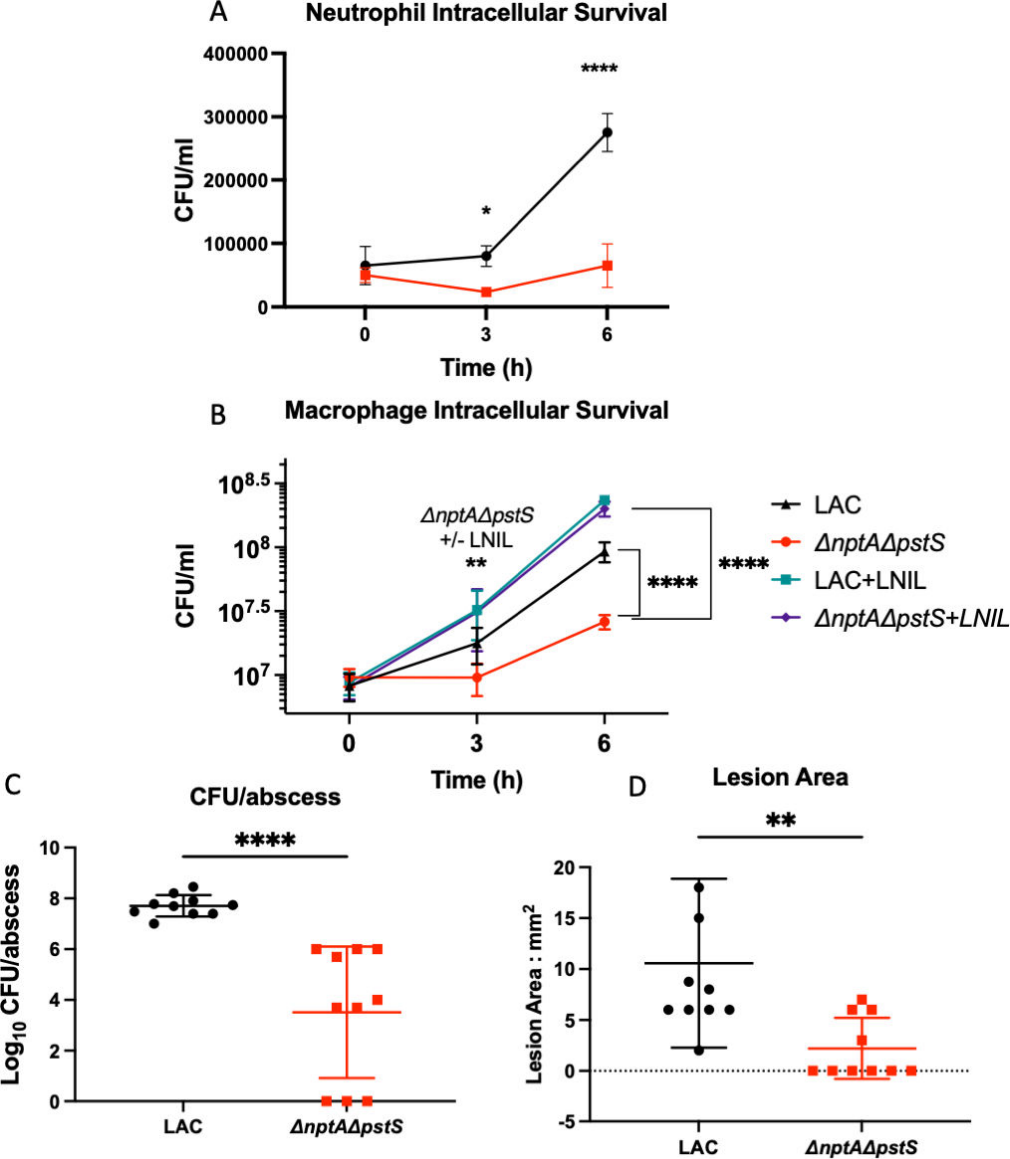
The Δ*nptA*Δ*pstS* double mutant is attenuated *in vivo*. Intracellular survival assays were performed on WT LAC and the Δ*nptA*Δ*pstS* mutant in neutrophils derived from MPRO cells (*n* = 4) (**A**) and RAW264.7 cells (*n* = 9) (**B**). In RAW264.7 macrophages, L-NIL, an inhibitor of iNOS, was added to determine the impact of NO on intracellular survival. 6–8-week-old C57BL/6J mice were subcutaneously infected with WT LAC and the Δ*nptA*Δ*pstS* mutant (*n* = 10) and assayed for CFU/abscess (**C**) and lesion area (**D**) at day 7 post-infection. Statistics: (**A**) two-way ANOVA with Sidak’s multiple comparisons, (**B**) two-way ANOVA with Tukey’s multiple comparisons, (**C and D**) unpaired *t*-tests. * = *P* < 0.05, ** = *P* < 0.01, **** = *P* < 0.0001.

To assess the role of these phosphate transporters coupled with NO· *in vivo*, we performed intracellular survival assays on WT *S. aureus* LAC and the Δ*nptA*Δ*pstS* isogenic mutant. We used promyelocytic cell line MPRO differentiated into neutrophils (Fig. 5A) and RAW264.7 macrophages (Fig. 5B). In each cell type, WT LAC starts to replicate after 3 h and has almost doubled its CFU count by 6 h. The Δ*nptA*Δ*pstS* mutant, however, never starts to replicate and has significantly lower CFU counts by 6 h. We were able to demonstrate that genetic complementation of the Δ*nptA*Δ*pstS* mutant with either *nptA* or *pstSCAB* was sufficient to restore growth of the double mutant to WT levels in RAW264.7 cells (Fig. S6). We treated the RAW264.7 macrophages with the iNOS inhibitor L-NIL, known to limit inflammatory NO· production. L-NIL treatment allowed for additional outgrowth of WT LAC at later timepoints (Fig. 5B). Interestingly, the addition of L-NIL also allowed for the outgrowth of Δ*nptA*Δ*pstS* to WT levels. This included significant growth over the untreated counterpart at both 3 and 6 h, indicating that NO· causes a significant impairment in Δ*nptA*Δ*pstS* growth in immune cells.

We also tested the ability of a Δ*nptA*Δ*pstS* mutant to cause infection in a skin and soft tissue infection model (SSTI). We subcutaneously infected WT C57BL/6J mice with 10^7^ CFU of WT LAC and the isogenic Δ*nptA*Δ*pstS* strain. We monitored the weight loss of the mice for 7 days and, at day 7, sacrificed the mice to measure lesion area and CFU/abscess. We found significant attenuation of the Δ*nptA*Δ*pstS* mutant in both abscess formation and CFU/abscess as compared to WT LAC, indicating a defect in virulence and survival of the Δ*nptA*Δ*pstS* mutant (Fig. 5C and D).

## DISCUSSION

Under NO· stress in a host, *S. aureus* must adapt rapidly and efficiently to a new metabolic state that is independent of the TCA cycle and ETC to survive. Our lab has characterized several aspects of this response—increased glucose transport, increased fermentation of pyruvate to lactate—and has now added another facet—alternative phosphate transport. The RNA-sequencing study published here aimed to identify aspects of the NO response that are transcriptionally dependent on glucose. The *pstSCAB* operon fell out as the most differentially regulated operon in the presence of glucose compared to its absence. Preliminary investigation into the genetic regulation of the *pstSCAB* system shows that CcpA, a carbon catabolism regulator, and PhoU, the regulatory protein downstream of the *pstSCAB* operon, are not responsible for this glucose-dependent regulation (Fig. S4). Additionally, *pstSCAB* is not regulated by Rex, the redox-sensing repressor that is responsible for the NO-dependent regulation of many NO·-inducible genes (16). Thus, further investigation into the genetic control of the *pstSCAB* operon is needed which, in tandem with *ldh*1, may reveal a global glucose-dependent regulator responsible for both *pstSCAB* and *ldh*1 expression during NO· stress. We can also test *nptA* in this analysis, as there is differential induction of *nptA* by glucose, though it is not differentially regulated under NO· stress.

Here, we expand upon why alkaline conditions necessitate one of these two phosphate transporters specifically. Previous reports showed their requirements in alkaline media, which we replicated here (19). We also show that upon extracellular alkaline stress, *S. aureus* does not defend its cytosolic pH. This likely reflects the fact that *S. aureus* rarely encounters alkaline conditions, as the surface of the skin is mildly acidic. In any event, when the extracellular pH is 8.5, the cytosolic pH of *S. aureus* will also be 8.5. We’ve previously shown that due to the ATP hydrolysis mode of the F_1_F_0_-ATPase during NO· stress, the cytosolic pH rises to ~8.5 as protons are extruded from the cell to maintain proton motive force (PMF) (15). In addition to contributing to PMF, this proton extrusion also raises the cytosolic pH to the optimum for all three lactate dehydrogenases, which are critical for full NO· resistance (27). We predicted that this rise in cytosolic pH would also necessitate one or more of the phosphate transporters required under alkaline conditions. Indeed, *S. aureus* requires either *pstSCAB* or *nptA* during NO· stress, likely due to the pH of the cytosol. The remaining phosphate transporter in the Δ*nptA*Δ*pstS* mutant is PitA, a PMF-dependent phosphate transporter that is highly dependent on pH. However, PitA also has the lowest affinity for inorganic phosphate of any phosphate transporter. When taken together with a change in PMF, a common factor in alkaline and NO· stress, this low affinity for inorganic phosphate is likely the reason for the inability of PitA to keep up with the phosphate transport needs of the cell. As a result, we see a strong growth defect in a strain that only contains PitA when these conditions are met.

The link between phosphate transport and glucose may not initially be evident until one looks closely at the process of glycolysis. The sixth step of glycolysis, mediated by GAPDH, involves the incorporation of inorganic phosphate into the GAP molecule, creating 1, 3-bisphosphoglycerate and NADH. The inorganic phosphate that was incorporated in this step is subsequently used to generate ATP from ADP in later steps of glycolysis, allowing for the incorporation of inorganic phosphate into the energy pool. Therefore, higher glycolytic flux in the cell requires more intracellular inorganic phosphate for efficient energy production.

Glycolysis is not the only metabolic process to integrate inorganic phosphate into cellular processes. Another way that inorganic phosphate is incorporated into the energy pool is via the ArcABCD system (28, 29). In the absence of glucose, *S. aureus* consumes amino acids. One of the primary amino acids consumed for energy is arginine, which is converted to citrulline via ArcA. The resulting citrulline is converted to ornithine and carbamyl phosphate by ArcB, a reaction that incorporates inorganic phosphate. The carbamyl phosphate molecule is used by ArcC, which transfers the phosphate to ADP-yielding ATP. This process is vital for the integration of inorganic phosphate into the ATP pool of the cell in the absence of glucose. This is reflected in our RNA-Seq screen as well*—arcC* can be seen in Fig. 1A as induced by NO· in casamino acids and pyruvate specifically. This indicates, again, the vital importance of inorganic phosphate and the energy pool under NO· stress, even in the absence of glucose.

Other metabolic genes of interest came up in the RNA-Seq screen. A 2,3-butanediol synthetic pathway (*alsS/aldC*) that has been documented as playing a role in NO· resistance was more highly upregulated in glucose by NO· than in casamino acids and pyruvate (30). We also see *hld*, the small toxin encoded in RNAIII, the expression of which has been demonstrated to be impacted by pyruvate (31). This is particularly interesting, however, because RNAIII appears to possibly be upregulated in our sample, despite the sample being taken at a low OD of 0.5. Finally, *lrgAB*, the two most differentially regulated genes under NO· stress in casamino acids and pyruvate, have recently been demonstrated to encode pyruvate transporters in both *Staphylococcus* and *Streptococcus* species (32, 33). If LrgA and LrgB are, in fact, upregulated here because they are transporting pyruvate into the cell; this could go a long way toward explaining how *S. aureus* can still resist NO· when glucose is absent, but pyruvate is present.

The field has clearly shown that *S. aureus* has evolved to resist NO·. This is specific to *S. aureus* and its role as a pathogen, as it does not see these levels of NO· unless it has perturbed the immune system. We have shown that *ldh1* was recently acquired by *S. aureus* and its most closely related species *Staphylococcus simiae* as compared to other coagulase-negative *Staphylococci* (CoNS). This is not necessarily true of *glcA* and *glcC*, which are important glucose transporters. While *S. simiae* does encode GlcA, it lacks a *glcC* paralog altogether (34). Similarly, *nptA* is absent from closely related species such as *Staphylococcus epidermidis* and *S. simiae* but is present only in *S. aureus* despite being found in many more divergent staphylococcal species (Fig. S1) (19). This study shows that this transporter, NptA, is important for growth under NO· stress, both *in vitro* and *in vivo*, and especially in an SSTI model and was reacquired by *S. aureus* likely because of the advantage this extra transporter confers. Other studies done with the Δ*nptA*Δ*pstS* mutant showed a more minor level of attenuation in a systemic infection model than we observed in a SSTI model (19). This may reflect the fact that *S. aureus*, as a pathogen, has evolved to persist in a skin infection, not necessarily in a systemic infection. However, from these studies, it is clear that, in response to host NO· and host glucose, *S. aureus* coordinates the expression of various glucose and phosphate transport systems as well as highly active fermentative enzymes to elicit a metabolic state that is compatible with inflamed host tissues replete with immune radicals such as NO·.

## MATERIALS AND METHODS

### Bacterial strains and growth

The strains used in this study are listed in Table 1. All mutant strains are derived from USA300 strain LAC. The Δ*nptA* strain was made as previously described, using allelic exchange via the plasmid pBTK* (10). Briefly, 1 kb regions from either side of the *nptA* gene were cloned into the pBTK* plasmid on either side of the kanamycin resistance cassette. This plasmid was isolated from *Escherichia coli* and transformed into *S. aureus* strain RN4220 (35). A lysate of phi-11 phage was created from this strain and used to move the modified pBTK* plasmid into LAC (36). The pBTK* plasmid was integrated into the LAC chromosome at the *nptA* locus via homologous recombination after the temperature-sensitive plasmid was exposed to temperatures of 43°C. Excision of the remainder of the plasmid was incited by the use of cyclosporin treatment. The Δ*pstS* transposon insertion mutation from the Nebraska Transposon Mutant Library from NARSA was transduced into LAC via phi-11 phage transduction. Phage transduction was also used to combine these two mutations into the Δ*nptA*Δ*pstS* mutant. The Δ*ccpA* mutant was previously described, and the Δ*phoU* and Δ*phoPR* mutants were also from the NARSA library. Complement plasmids were created by amplifying the promotor region of either *nptA* or *pstSCAB* along with the gene(s) and cloned into the plasmid pOS1. The plasmid was transformed into *S. aureus* strain RN4220 and subsequently transduced into the target strains.

**TABLE 1:** Strains used in this study

Strain name	Strain description	Source
WT LAC	Methicillin-resistant clinical *S. aureus* isolate	
AR1754	*S. aureus* LAC *ΔnptA::*Km	This study
AR1755	*S. aureus* LAC *ΔpstS::*Tn-Erm	This study
AR1756	*S. aureus* LAC *ΔnptA::*Km *ΔpstS::*Tn-Erm	This study
AR1749	*S. aureus* LAC Δ*ccpA::*Kan	
NE1316	*S. aureus* JE2 Δ*phoU*::Tn	NARSA
NE1486	*S. aureus* JE2 Δ*phoP*::Tn	NARSA
AR1787	Δ*pstS*::Tn; Δ*nptA*::km; plgt::*nptA*	This Study
AR1790	Δ*pstS*::Tn; Δ*nptA*::km; pOS1::*pstSCAB*	This Study

Overnight cultures were grown in brain heart infusion (BHI) (BD Biosciences). For RNA-Seq experiments and qRT-PCR confirmation, strains were grown in a chemically defined minimal media, known as PN media, supplemented with 0.5% glucose or 0.5% casamino acids and 0.5% pyruvate in a 50 mL culture volume in a 500 mL flask to ensure aeration (37). When stated, a modified version of PN was used with a defined phosphate concentration. Briefly, the PN salts were removed and replaced with a Tris buffer solution of pH 7.4. The media was then supplemented with 10 mM or 0.1 mM K_2_HPO_4_ (high- and low-phosphate conditions) and 10 mM NaCl. Cultures were grown in this media at an aeration ratio of 1:10 and an inoculum ratio of 1:200 from overnight cultures grown in BHI. For growth curves, a 1:200 inoculum ratio was used in a 200 µL culture in a 96-well plate and grown at 37°C shaking in a BioTek Synergy H1 plate reader. When stated, 10 mM DETA-NO (Sigma) was added to cultures. Cultures containing complementation plasmids were grown in 10 µM chloramphenicol.

### RNA extraction

RNA extractions were performed as previously described (16). Briefly, 25 mL of culture was quenched with 25 mL of ice-cold ethanol:acetone. Samples were stored at −80°C for no more than 4 days. On extraction, samples were thawed at room temperature and pelleted at 5,000 × g for 10 minutes. Supernatant was discarded, and pellets were dried at room temperature, then resuspended in 100 µL of TE. These resuspensions were freeze-thawed in an ethanol-dry ice bath three times, thawing at 60°C each time. Samples were then bead-beat for 1 minute, rested on ice for 5 minutes. 650 µL of lysis buffer was added to the samples, and bead-beating was repeated. Lysis tubes were then centrifuged at 13,000 × g for 2 minutes, and supernatants were removed and combined with an equal volume of 70% ethanol. This mixture was processed with the Invitrogen Pure-link Mini RNA extraction kit. Samples were treated with DNAse-1 (NEB) for 1 h, then repurified using the Invitrogen Pure-link Mini RNA kit. The samples were then quantified.

### RNA-sequencing and analysis

The RNA extracted as above was sent to the University of Pittsburgh Health Sciences Sequencing Core at Children’s Hospital of Pittsburgh. The stranded total RNA library was prepared using the TruSeq Total RNA kit (Illumina). 300 ng of RNA was depleted for rRNA using bacterial target for rRNA capture, then cleaned up with AMPureXP beads, and then fragmented. Random primers initiate first and second-strand cDNA synthesis. First-strand cDNA synthesis used SuperScript IV. The adenylation of 3’ ends was followed by adapter ligation and 12 cycles of library amplification with indexing. The amplified library was cleaned up with 45 µL AMPureXP beads. Sequencing was performed on a NextSeq500, with a MidOutput 150 Flowcell. The read length was 150 bp, and the loading concentration was 1.8 pM. Demultiplexing and adapter sequence trimming were performed by the Core. Samples were analyzed via alignment to the *S. aureus* LAC genome using Geneious v.8. The data from this RNA-Seq experiment are presented in Table S1 and are publicly available on BV-BRC.

### RT-PCR

50 ng of purified RNA was used in a qRT-PCR reaction as according to the manufacturer’s instructions for the Power SYBR green RNA-to C^t^ 1-step kit (Applied Biosystems). An iQ5 machine was used for qRT-PCR, and the coordinating iQ5 software was used to determine the C_t_. The ΔΔC_t_ was found using *rpoD* as a reference gene. Primers used are listed in Table 2.

**TABLE 2:** Primers used in this study

Primer	Sequence
NptA_5'.1A	GGGGGATCCTGTAAACGTGACCCACTTGC
NptA_5'.1B	GGGGGATCCCTTCTGTAACCGACATTTCC
NptA_3'.1A	GGGGAATTCTCCAACATTCATGGGTTGGC
NptA_3'.1B	GGGGAATTCGGTTATACAGCATTGCAGGC
rpoD_RT.1A	AACTGAATCCAAGTGATCTTAGTG
rpoD_RT.1B	TCATCACCTTGTTCAATACGTTTG
ldh1_RT.1A	AAAACATGCCACACCATATTCTCC
ldh1_RT.1B	TACTAAATCTAAACGTGTTTCTCC
PstS_RT1.A	TGGCTCATCAACAGTAGCAC
PstS_RT1.B	AAACCAGCACCTGTACCAGC
NptA_Rt1.A	TTCAAGCATCAGCAGGAGAC
NptA_Rt1.B	GTCGTACCTGAACTACTTTG
PitA_RT1.A	ATGGATTCCATGATACAGCC
PitA_RT1.B	TAAAGTTCATCACTGCTGCC
NptA.1A	GGGCATATGGAGGTGAAATAATGGAAATGTCGG
NptA.1B	GGGGGATCCGGCTTTTTACATGCATCTGAATCTC
PstSCAB.1A	GGGGGATCCTAGGGATTTATGTCCCAGCC
PstSCAB.1B	GGGGGATCCGTTGCTCCTGATATCGTTGTC

### Phosphate quantification

Phosphate in CDM-G and CDM-CP was quantified using a Biomol Green assay (Enzo Life Sciences) following the manufacturer’s instructions. Standards provided in the kit were used to quantify the amount of phosphate present in these media.

### Intracellular pH and ATP experiments

Intracellular pH of LAC grown in CDM-G at varying extracellular pH was assayed using the pHrodo Red AM Intracellular pH Indicator Kit (ThermoFisher). Cells were grown to an OD of 0.2 and then 200 µL of the sample was washed with HEPES buffer at pH 7.4 and subsequently stained 50 nM pHrodo Red AM staining solution for 30 minutes at room temperature. Samples were washed with HEPES buffer and read for fluorescence on a BioTek Synergy H1 plate reader. A standard curve of samples treated with 10 µM valinomycin/nigericin at pH levels 4.5, 5.5, 6.5, and 7.5 was used to convert fluorescence into pH.

Intracellular ATP was measured using the BacTiter-Glo kit (Promega). Briefly, cultures were grown in phosphate-limiting CDM-G at pH 7.4, 8.5, or in the presence of DETA-NO. 100 µL samples were taken hourly and mixed with 100 µL of BacTiter-Glo reagent. The mixture was incubated for 5 minutes and then luminescence was determined using a BioTek Synergy H1 plate reader. The readings for identical ODs were compared to account for differing growth rates.

### Preparation of bacterial strains for cell culture infections

*S. aureus* LAC, the isogenic Δ*nptA*Δ*pstS* mutant, and complementation strains were grown overnight at 37°C in BHI. Cultures were diluted 1:200 in fresh BHI and grown for another 4 h at 37°C, harvested, and washed two times with PBS. OD _600_ was measured and adjusted to ensure a multiplicity of infection (MOI) of 10:1. The required bacteria were opsonized with an equal volume of normal mouse serum for 20 minutes at 37°C followed by final dilution into infection media. Bacterial CFU were enumerated at this stage to ensure the correct MOI.

### Bacterial survival in RAW 264.7 cells

RAW 264.7 cells (ATCC TIB-71) were cultured in RPMI 1640 (Gibco 11875-093) containing 1 mM sodium pyruvate and 10% fetal bovine serum (FBS) at 5% CO_2_. Cells were plated in 12-well plates with 10^6^ cells/well for 12–16 h. On the day of infection, cells were treated with 100 ng/mL lipopolysaccharide (LPS) and 20 ng/mL IFNγ for 6 h followed by three PBS washes. Cells were overlaid with opsonized LAC, the Δ*nptA*Δ*pstS* mutant, or complemented strains containing RPMI minus FBS and plates centrifuged at 200 × g for 5 minutes to allow efficient bacterial attachment. Plates were incubated at 37°C with 5% CO_2_ for 30 minutes. Wells were washed three times with PBS and incubated with RPMI containing 20 µg/mL gentamicin for 1 h. Cells were washed once with PBS and either incubated further or lysed for the 0-h time point. For lysis and CFU analysis at every time point, wells were incubated for 5 minutes with 1% Triton X-100, serially diluted, and plated on BHI agar. Complementation strains were plated on BHI agar + 10 µM chloramphenicol. Colonies were counted the next day. For L-NIL treatment, 100 µM L-NIL was added to the LPS IFN treatment wells, and the presence of L-NIL was maintained in the media at all stages till cell lysis.

### Bacterial survival in MPRO cells

MPRO cells (ATCC CRL-11422) were cultured in IMDM (Gibco 12440-046) containing 20% heat-inactivated horse serum and differentiated in culture media containing 10 µM ATRA for 72 h. Differentiated MPRO cells were distributed at 10^6^ cells/tube and treated with 100 ng/mL LPS and 20 ng/mL IFNγ for 6 h. Cells were washed by centrifugation at 200 × g for 5 minutes and incubated with opsonized LAC or the Δ*nptA*Δ*pstS* mutant or complementation strains in IMDM minus horse serum for 30 minutes at 37°C and 5% CO_2_. Cells were washed three times with PBS and incubated with IMDM containing 20 µg/mL gentamicin for 1 h. Cell lysis and CFU analysis were performed as described above.

### Animal infections

*S. aureus* LAC and the isogenic Δ*nptA*Δ*pstS* mutant were grown for 12–16 h at 37°C in BHI. Cultures were diluted to 1:200 in fresh BHI and grown till OD_600_ reached 2.0. 1 mL of each culture was harvested and washed twice with PBS. Bacterial pellets were reconstituted in 200 µL of PBS and serially diluted till 10^10^ dilution and plated on BHI agar. Colonies were counted the next day, while the reconstituted bacterial cultures were stored at 4°C. Bacterial suspensions were adjusted to 5 × 10^8^/mL based on CFU enumeration.

Both male and female, 6–8 weeks old C57BL/6J mice weighing 20–25 g were used in this study. Mice were obtained from Jackson laboratories and housed with 14 h light cycles. On the day of infection, mice were weighed, and 12× body weight in µL of Avertin was administered via intraperitoneal injection. The dorsal left flank of each animal was shaved, and 20 µL of the bacterial suspension was injected subcutaneously using a 26G needle. Animals were monitored every day. On day 7, the mice were euthanized in a CO_2_ chamber followed by cervical dislocation. The abscesses were measured, excised, and homogenized in PBS followed by serial dilution and plating on BHI agar for CFU enumeration.

## SUPPLEMENTAL MATERIAL

Fig. S1 mbio.02451-23-s0001.tif NptA phylogeny.

Fig. S2 mbio.02451-23-s0002.tif RNA-seq heat map.

Fig. S3 mbio.02451-23-s0003.tif Supplemental Q RT-PCR.

Fig. S4 mbio.02451-23-s0004.tif Regulator screen.

Fig. S5 mbio.02451-23-s0005.tif pH and NO complementation.

Fig. S6 mbio.02451-23-s0006.tif Intracellular survival complementation.

Table S1 mbio.02451-23-s0007.xlsx RNA-seq results.

ASM does not own the copyrights to Supplemental Material that may be linked to, or accessed through, an article. The authors have granted ASM a non-exclusive, world-wide license to publish the Supplemental Material files. Please contact the corresponding author directly for reuse.
