# Supplementary figures and images for: Specialized phosphate transport is essential for Staphylococcus aureus nitric oxide resistance
Source: mBio. 2023 Nov 8;14(6):e02451-23. doi: 10.1128/mbio.02451-23 (PMC10746193; doi:10.1128/mbio.02451-23)

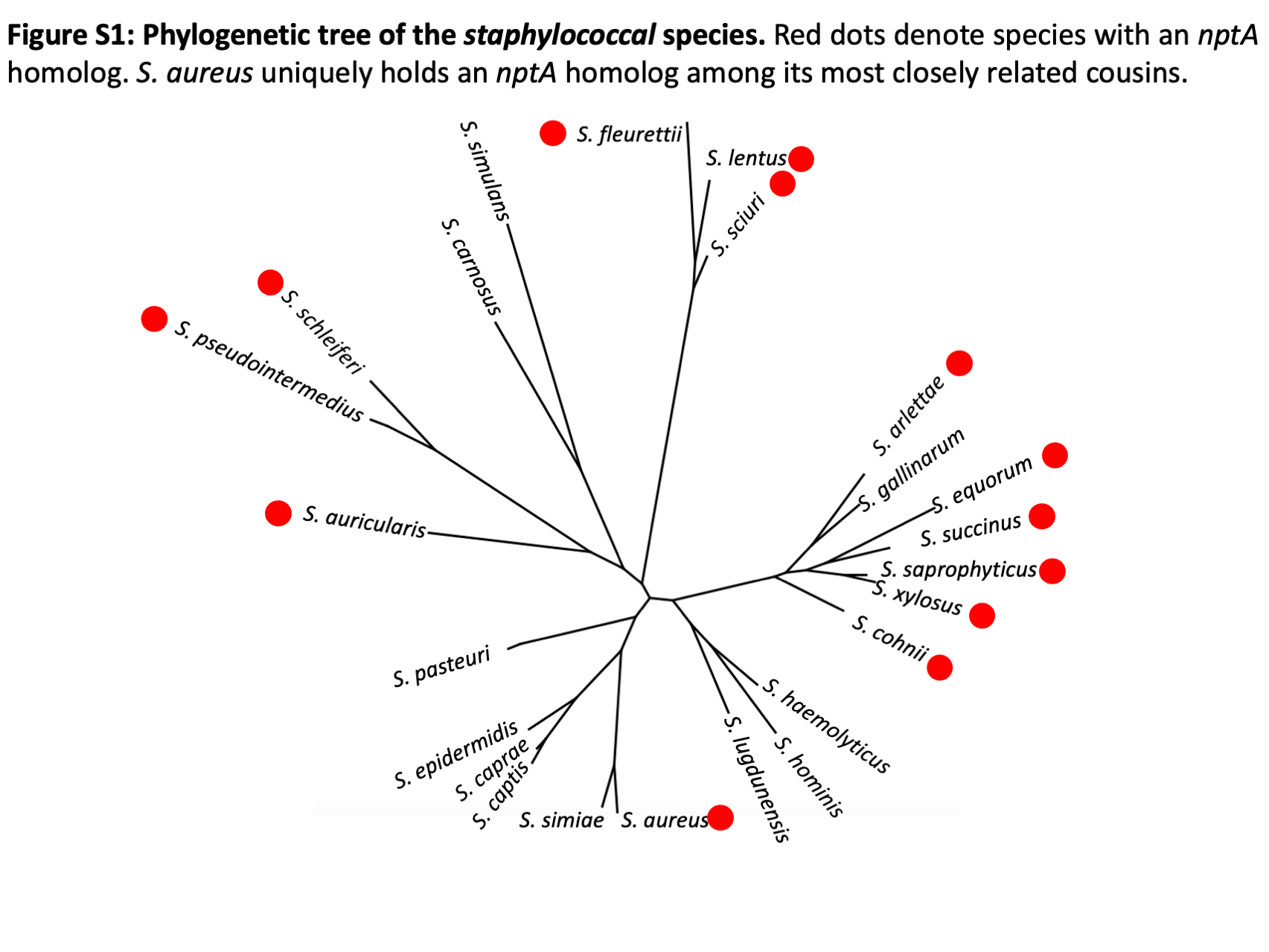

Supplement: Fig. S1 — NptA phylogeny. [file mbio.02451-23-s0001.tif]

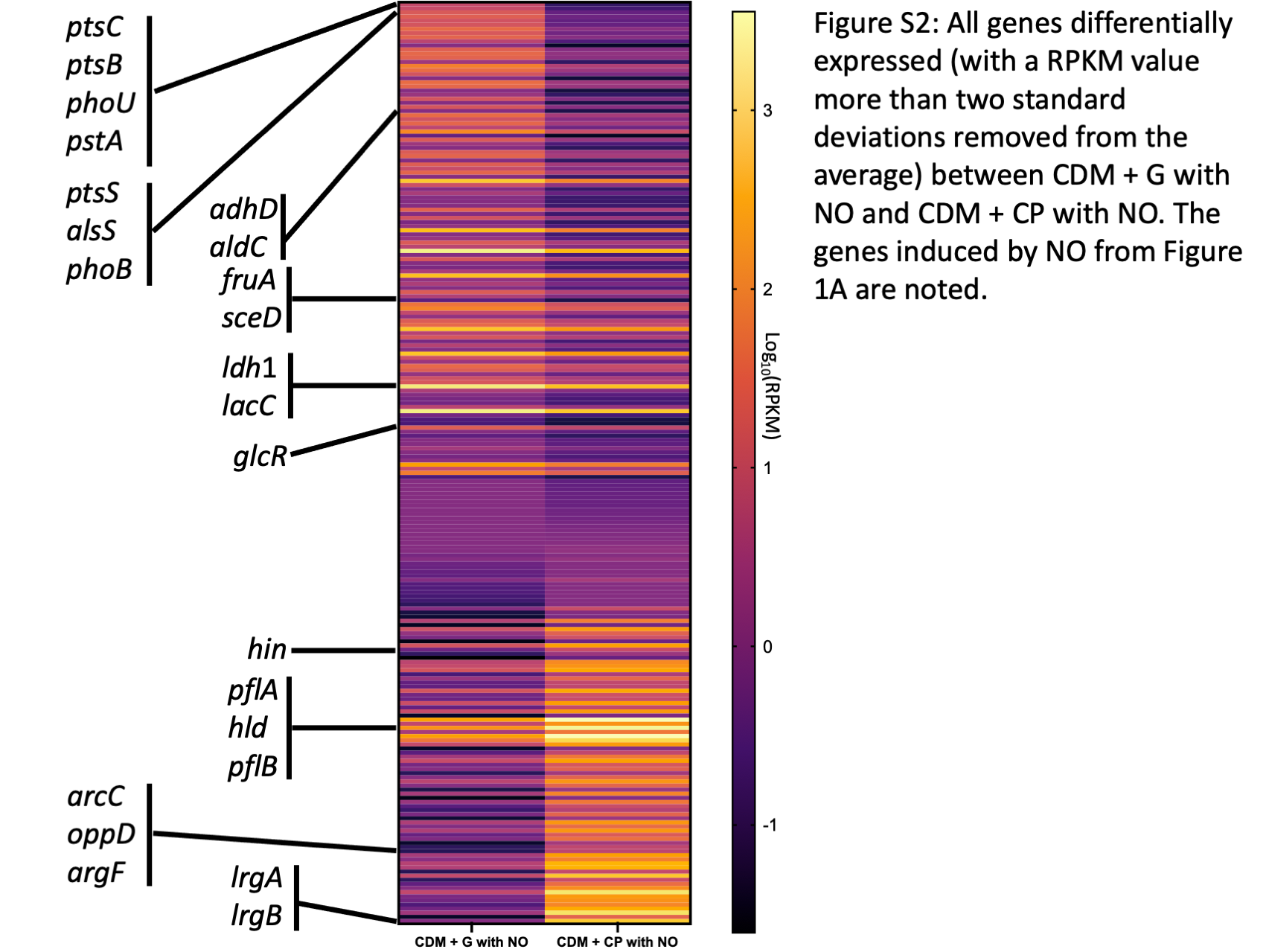

Supplement: Fig. S2 — RNA-seq heat map. [file mbio.02451-23-s0002.tif]

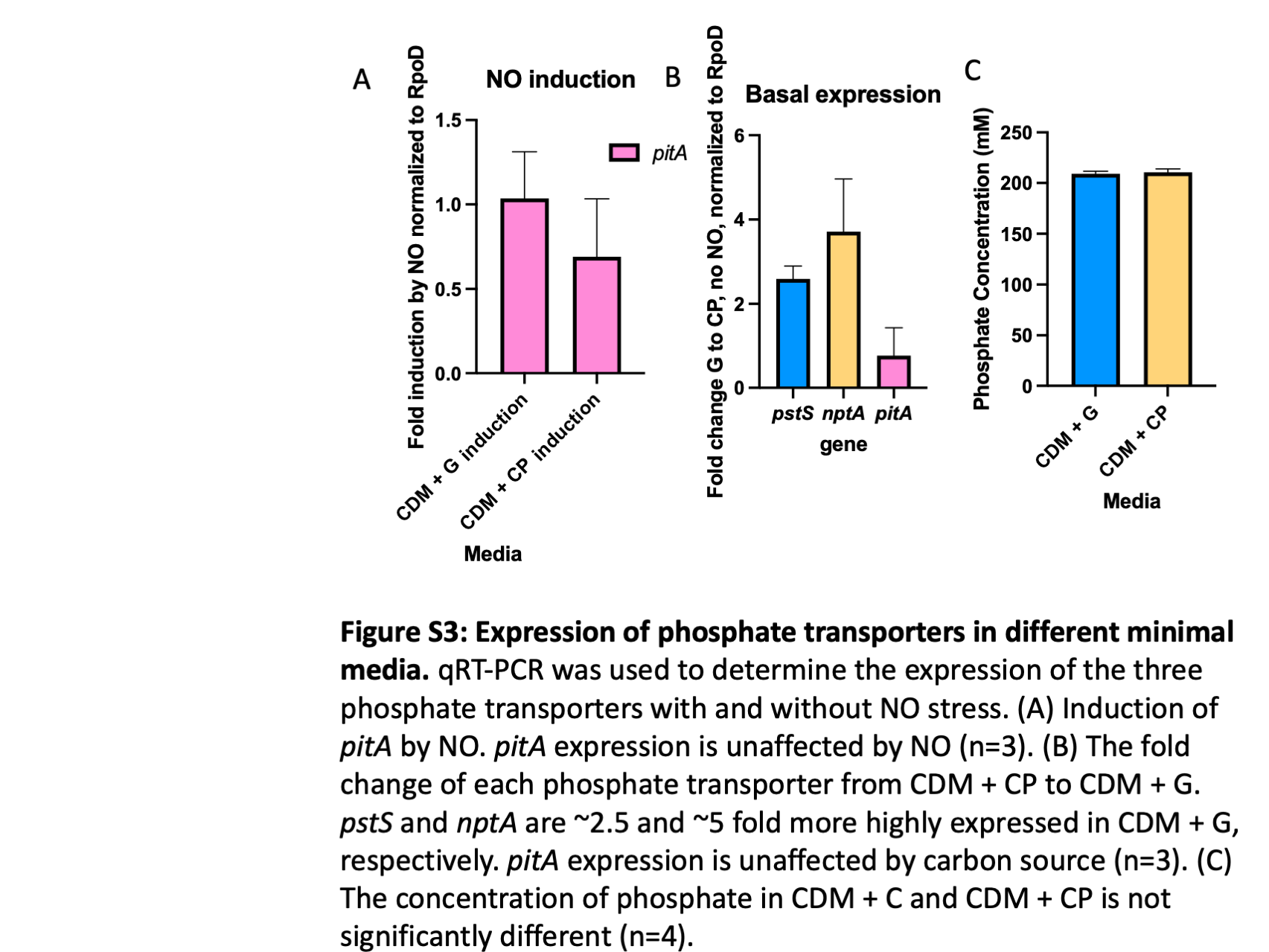

Supplement: Fig. S3 — Supplemental Q RT-PCR. [file mbio.02451-23-s0003.tif]

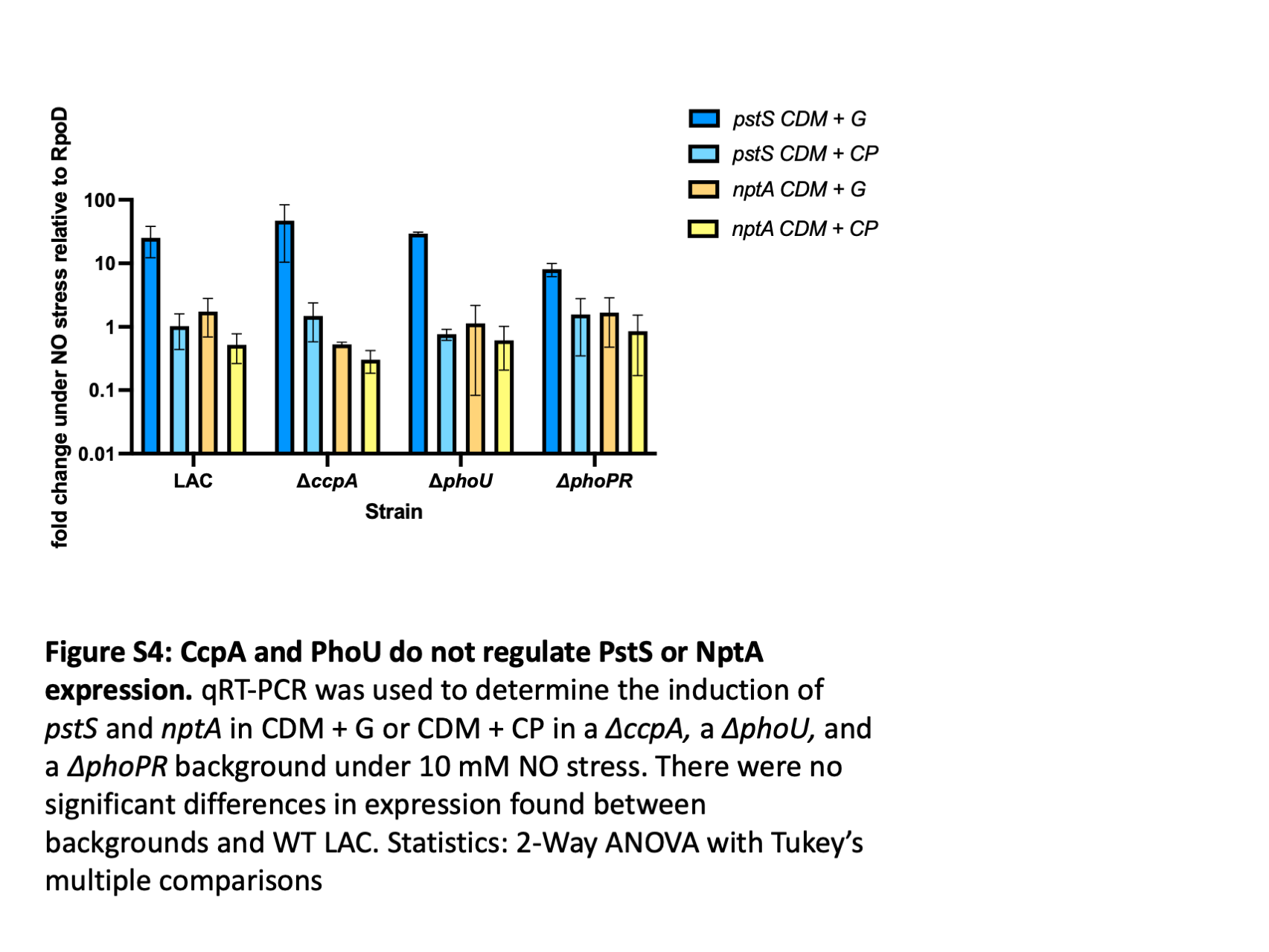

Supplement: Fig. S4 — Regulator screen. [file mbio.02451-23-s0004.tif]

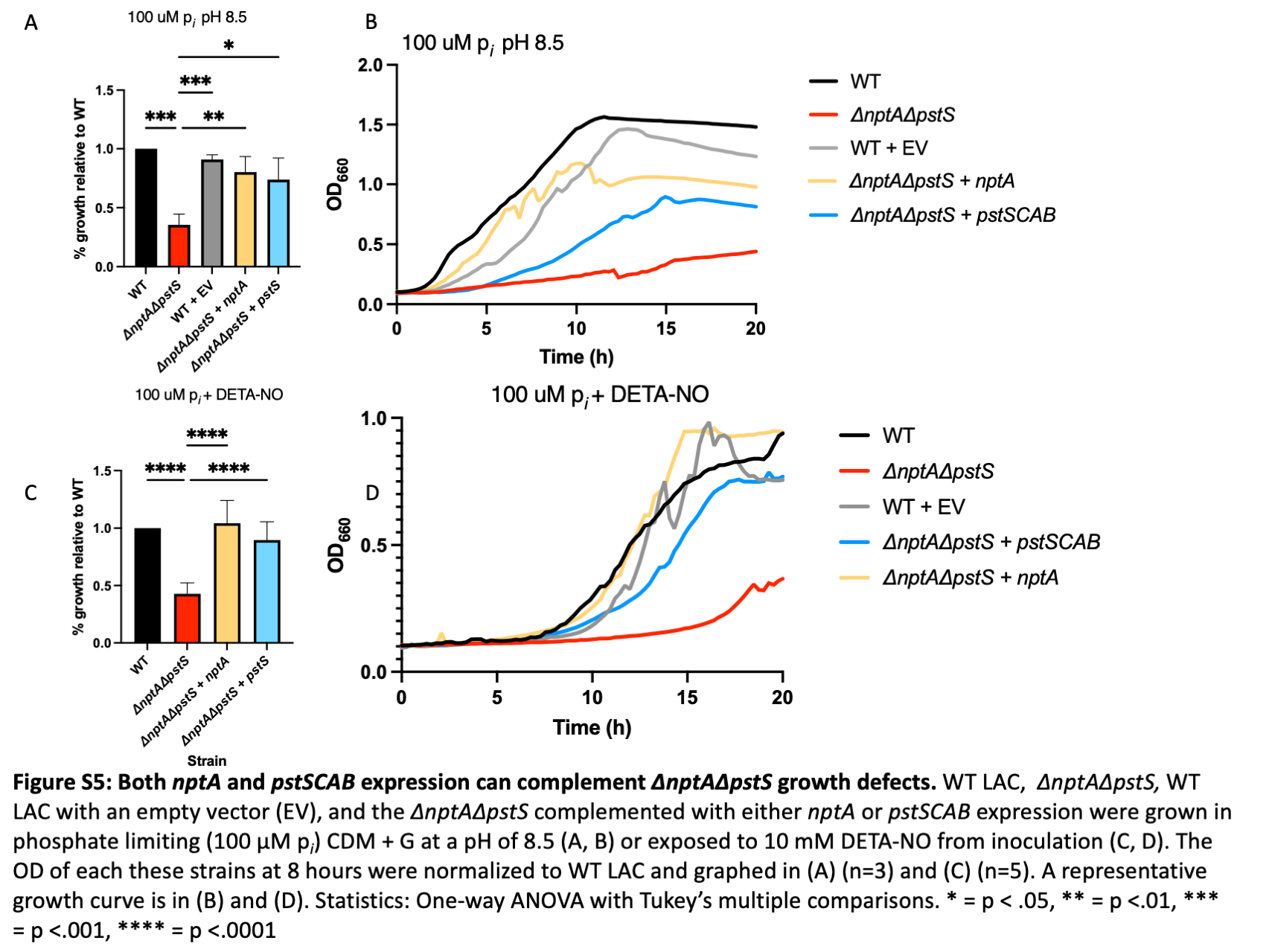

Supplement: Fig. S5 — pH and NO complementation. [file mbio.02451-23-s0005.tif]

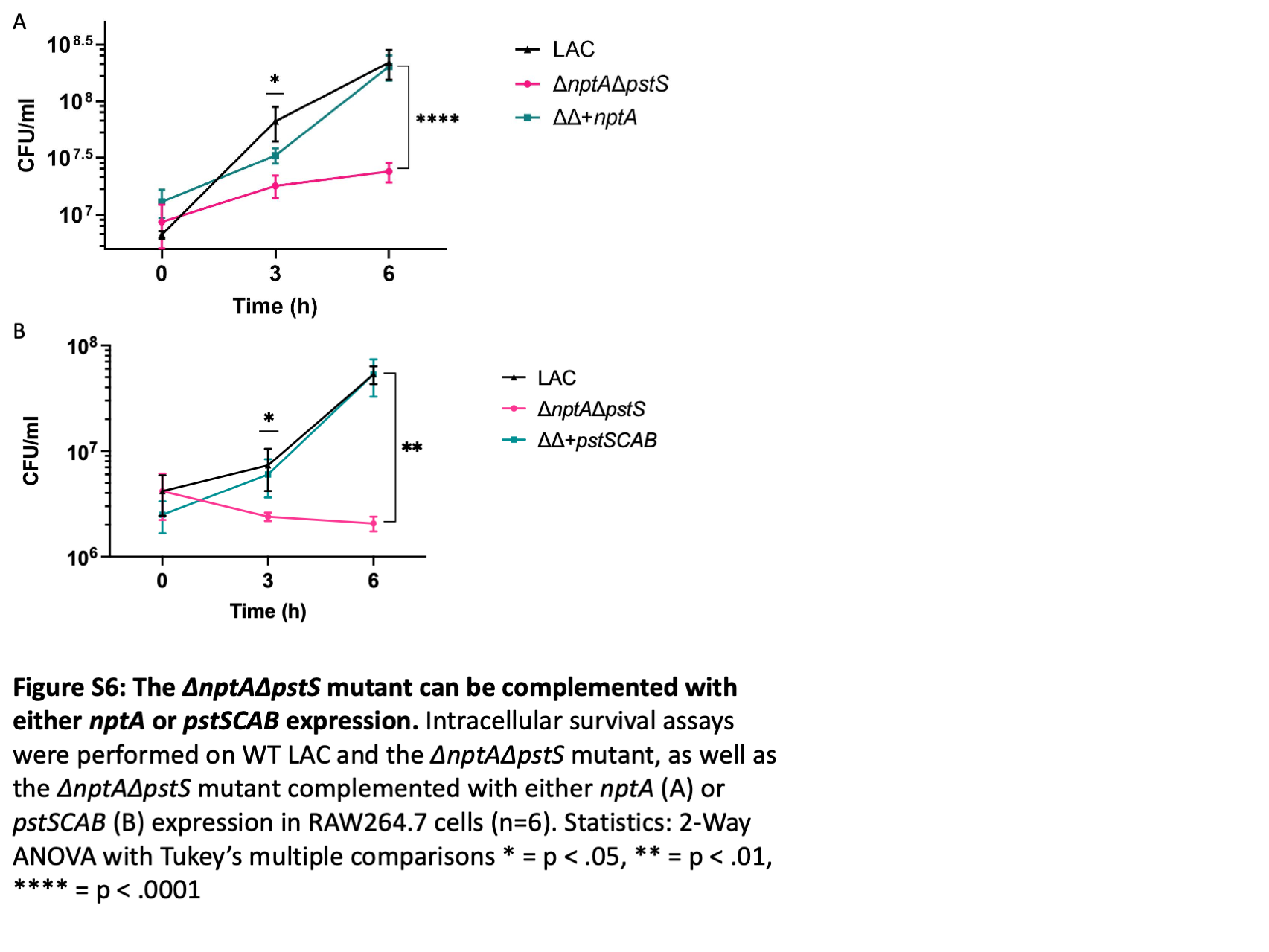

Supplement: Fig. S6 — Intracellular survival complementation. [file mbio.02451-23-s0006.tif]
